# t2prhd: a tool to study the patterns of repeat evolution

**DOI:** 10.1186/1471-2105-9-27

**Published:** 2008-01-18

**Authors:** Botond Sipos, Kálmán Somogyi, István Andó, Zsolt Pénzes

**Affiliations:** 1 Institute of Genetics, Biological Research Center of the Hungarian Academy of Sciences, P.O. Box 521, H-6701 Szeged, Hungary; 2 Department of Ecology, University of Szeged, Egyetem street 2, H-6721, Szeged, Hungary

## Abstract

**Background:**

The models developed to characterize the evolution of multigene families (such as the birth-and-death and the concerted models) have also been applied on the level of sequence repeats inside a gene/protein. Phylogenetic reconstruction is the method of choice to study the evolution of gene families and also sequence repeats in the light of these models. The characterization of the gene family evolution in view of the evolutionary models is done by the evaluation of the clustering of the sequences with the originating loci in mind. As the locus represents positional information, it is straightforward that in the case of the repeats the exact position in the sequence should be used, as the simple numbering according to repeat order can be misleading.

**Results:**

We have developed a novel rapid visual approach to study repeat evolution, that takes into account the exact repeat position in a sequence. The "pairwise repeat homology diagram" visualizes sequence repeats detected by a profile HMM in a pair of sequences and highlights their homology relations inferred by a phylogenetic tree. The method is implemented in a Perl script (t2prhd) available for downloading at  and is also accessible as an online tool at . The power of the method is demonstrated on the EGF-like and fibronectin-III-like (Fn-III) domain repeats of three selected mammalian Tenascin sequences.

**Conclusion:**

Although pairwise repeat homology diagrams do not carry all the information provided by the phylogenetic tree, they allow a rapid and intuitive assessment of repeat evolution. We believe, that t2prhd is a helpful tool with which to study the pattern of repeat evolution. This method can be particularly useful in cases of large datasets (such as large gene families), as the command line interface makes it possible to automate the generation of pairwise repeat homology diagrams with the aid of scripts.

## Background

The conceptual models developed to explain the evolution of multigene families [1,2] have also been applied to sequence repeats inside a gene/protein. Studies of repeat evolution [3-9] have often revealed complex patterns: some repeats may evolve in concert, subject to homogenization, while other repeats may maintain their sequence identity, which is more consistent with birth-and-death and/or divergent evolution. Phylogenetic reconstruction is an established approach with which to study the mode of evolution of multigene families [10-14] and repeats [3-9]. In the analysis of closely related species, the association of genes on the same locus (orthologues) on phylogenetic trees suggests independent evolution of the respective members, as expected for the birth-and-death model. On the contrary, the association of paralogues from the same species suggests homogenization, which is consistent with the concerted model. The application of the same logic to repeats poses a number of problems. Studies on the evolution of multigene families by phylogenetic methods make use of a prior concept of homology defined by the locus. As the term "locus" denotes genomic position, it follows that the exact repeat position in the full sequence (starting and ending positions) should be used to identify them. This approach has seldom been used in the previous studies, probably because it complicates the interpretation of the phylogenetic trees. Instead, a simple numbering according to repeat order is often applied, although this can be misleading: if a repeat is not detected by the selected method (for example the profile Hidden Markov Model (HMM) method) in one of the compared sequences, the ordinal numbers of the following repeats will be shifted by one and hence the analysis will become laborious.

## Results and Discussion

To simplify the analyses of repeat evolution, a novel rapid visual method has been developed to highlight the relationships of repeats detected in a pair of sequences by the profile HMM method. The primary aim of the application is the analysis of tandem amino acid sequence repeats but DNA sequences can also be used with a profile HMM trained with DNA sequences. When DNA sequences are used, the script does not consider the reverse complement of the sequences, so the usefulness of this application in a genomic context (for example in the case of transportable elements) might be limited, though the implementation can be easily extended to handle this kind of analysis.

The "pairwise repeat homology diagram" visualizes repeats in a simple scheme, together with their homology relations (orthology and paralogy) inferred by a phylogenetic tree, providing an intuitive way to analyse the patterns of repeat evolution. This visualization also facilitates a survey of the sequence structure (size, linker sequences and non-repetitive regions). Regions in which consecutive repeats are connected with those in the other sequence forming a ladder-like pattern are most likely to have evolved independently following the birth-and-death and/or divergent process. On the contrary, in regions where repeats have only internal or no identified connections, repeats possibly evolve in concert. The patterns demonstrated by internal connections can reveal units of concerted evolution or recent internal duplications. It should be taken into account, however, that if there are clades formed by more than two identical sequences, the branching pattern of such clades, and hence the identified relation, is arbitrary and uninformative in this kind of analysis.

### Implementation

The method is implemented in a Perl script with command line interface (t2prhd, standing for "tree to pairwise repeat homology diagram"). The repeats are first identified with *hmmsearch *from the *HMMER *package [15] and a profile HMM specified by the user. Raw search results are parsed by using *BioPerl *[16] modules and the extracted repeats are aligned with *hmmalign*. The resulting alignment is converted into Fasta and sequencial PHYLIP formats and a phylogenetic tree is built by using *CLUSTAL W *[17] (with default parameters) or by using *PhyML *[18]. The script reads in the resulting tree as a *Bio::Tree::TreeI *object. After getting the list of all leaf nodes it finds the "sister leaf nodes" (leaf node pairs having the same ancestor) by an algorithm that for *n *leaf nodes (repeats) needs in the best case $\frac{n}{2}$ and in the worst case *n *iterations. So, the asymptotic upper bound for the time complexity of this algorithm is *O*(*n*) and the asymptotic lower bound is Ω($\frac{n}{2}$). These sister leaf nodes have a most recent common ancestor as indicated by the tree and accordingly we regard them as unambiguously identified homologues.

The script creates an SVG (Scalable Vector Graphics) file containing the diagram by using the *XML::Writer *module and saves the outputs and logs produced by the external applications. Optionally, it can also generate a LaTeX output. The identified homology relations are represented by lines or arcs connecting the respective repeats: blue lines are drawn between repeats from different sequences (orthology), and brown arcs in cases of internal relations (paralogy). The colour intensity of the connecting lines is a function of the patristic distance between the respective leaf nodes (*d*) divided by the total tree length (*T*): ${\left(1-\frac{d}{T}\right)}^{w}$. The default value of the "colour gradient parameter" *w *is 1 (linear colour scale), but by setting this parameter the colour scale can be tuned so that one can discriminate between close distance values. To make the interpretation of the colour scale easier a legend is drawn.

The generated SVG file can be viewed by *Firefox *1.5 [19] or higher as an example and can be rasterized (and also viewed) using the *Batik *toolkit [20] or any other image editor capable to handle the SVG format. The generated LaTeX file should be processed by *pdflatex*, the *pgf/TikZ*, *fancyhdr*, *xcolor *and *fullpage *packages are required. The script has a manual page embedded in POD format.

The script is also accessible as an online tool [21] (with *CLUSTAL W *back-end only) through a web interface created by using the *Pise *form generator [22]. The real power of this visual method is manifested in studies with large data sets, where the analysis of numerous or large trees would be highly laborious. In these cases, it is very advantageous that the command line interface enables the use of the scripts to automate the diagram generation.

### Example

Tenascins are extracellular matrix glycoproteins containing regions of repeated EGF-like and fibronectin-III-like (Fn-III) domains. The evolution of these repeated domains in mammalian Tenascins has been studied in detail by means of phylogenetic and other methods [8]. To illustrate the power of the method, we generated pairwise repeat homology diagrams with three selected protein sequences. The protein sequences corresponding to the DNA sequences studied by Hughes [8] were used (abbreviations and GenBank accession numbers in parentheses): human Tenascin X (TXH, [GenBank: AAB47488.1]), human Tenascin C (TCH, [GenBank: CAA39628.1]) and murine Tenascin X (TXM, [GenBank: AAB82015.1]). The profile HMM files were downloaded from Pfam (EGF-like: PF00008.17, Fn-III: PF00041.11).

**Figure 1:**
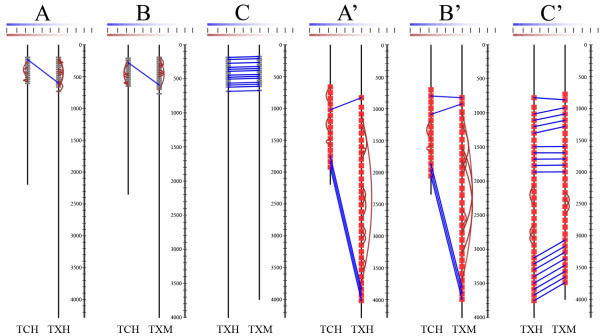
**Pairwise repeat homology diagrams of selected Tenascin proteins (SVG outputs)**. The diagrams were produced with the default PhyML parameters of the script (WAG+Γ+I, 4 gamma categories, gamma parameter and proportion of invariable sites estimated by ML, BIONJ starting tree) and with colour gradient paramater *w *set to 2. The repeats (EGF on A, B and C; Fn-III on A', B' and C') are indicated by red rectangles in the protein sequence schemes (N-termini on top). The identified homology relations are represented by lines or arcs connecting the respective repeats (blue lines between repeats of different protein sequences (orthology), and brown arcs in case of internal relations (paralogy)). The colour scale bar demonstrates the colour intensities as a function of the patristic distance between the respective clades divided by the total tree length. The lines represent the values between 0 and 1 by units of 0.1. The sequence positions are shown on the right of each scheme. Abbreviations: TCH: *Homo sapiens *Tenascin C; TXH: *Homo sapiens *Tenascin X and TXM: *Mus musculus *Tenascin X.

Hughes [8] concluded that EGF domain repeats underwent homogenization within each Tenascin gene after duplication, but remained conserved after the divergence of rodents and primates. The same conclusions can be drawn after the evaluation of the diagrams generated with paralogous (Figure 1A and 1B) and orthologous (Figure 1C) sequence pairs. Our diagrams are also in accordance with the conclusions of Hughes regarding the evolution of Fn-III type domain repeats. These repeats can be divided into three categories. The last three C-terminal repeats demonstrate conservation since the duplication of the Tenascin X and Tenascin C genes (Figure 1A', B' and 1C'). Other repeats became homogenized within each gene subsequent to gene duplication, but have remained conserved following the divergence of primates and rodents (Figure 1C'). The repeats of the third category have evolved in a concerted fashion in rodent and primate lineages since their divergence (Figure 1C').

## Conclusion

**Figure 2:**
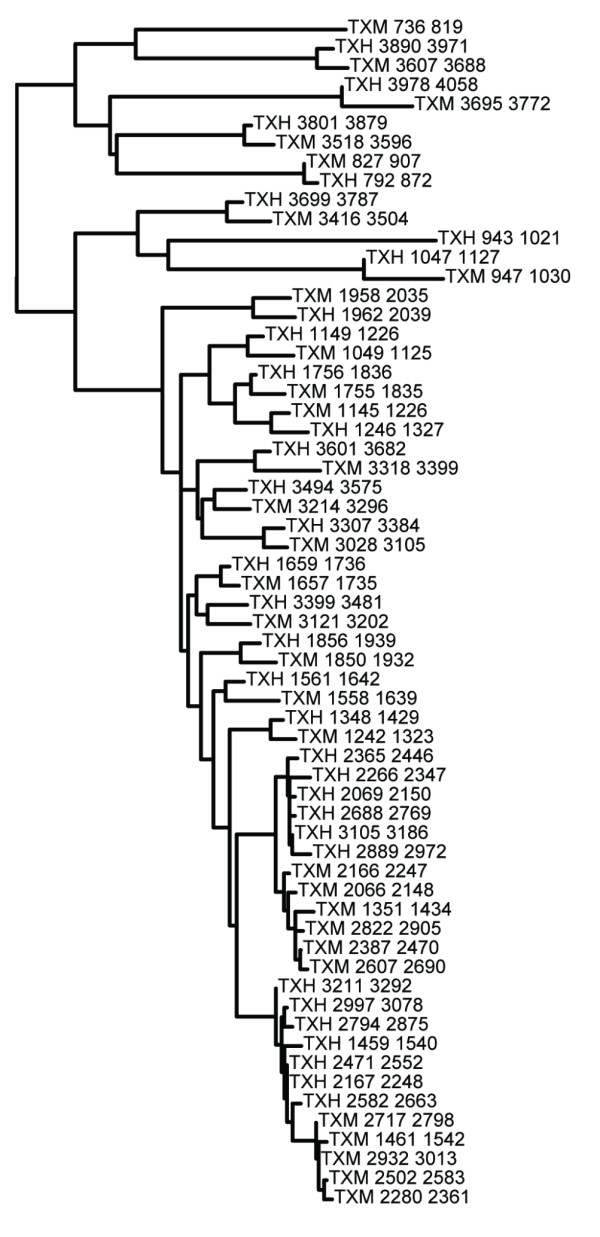
**Phylogenetic tree of Fn-III repeats of Tenascin X proteins of *Homo sapiens *and *Mus musculus***. Maximum Likelihood tree produced during the analysis of human versus murine Tenascin X sequences with Fn-III profile HMM (diagram on Figure 1C'). Repeats are identified by starting and ending positions. Abbreviations: see Figure 1.

Although pairwise repeat homology diagrams do not carry all the information about the phylogenetic tree on which they are based, by visualizing the exact positions of the repeats and the homology relations, they permit a rapid and intuitive assessment of the patterns of repeat evolution (compare Figure 1C' with Figure 2). These features make t2prhd a powerful tool, especially in cases of massive datasets, as in studies of repeat evolution in large gene families.

## Availability and requirements

• **Project name: **t2prhd

• **Project home page: **

• **Online access: **

• **Operating system(s): **OS Independent (Written in an interpreted language)

• **Programming language: **Perl

• **Other requirements: **Perl version 5.8.8 or higher with the standard modules, *BioPerl *modules (version 1.4.0 or higher), *XML::Writer *module, *HMMER *package 2.3.2 or higher, *CLUSTAL W *version 1.83 and/or *PhyML *version 2.4.4 or higher

• **License: **GNU General Public License

• **Any restrictions to use by non-academics: **none

## Authors' contributions

BS, KS and ZP developed the approach. BS wrote the program and the manual and created the website. ZP reviewed the code and the manual and tested the program functionality. BS and KS wrote the manuscript. IA and ZP conceived and coordinated the project and refined the manuscript.
